# Recruitment of an Activated Gene to the Yeast Nuclear Pore Complex Requires Sumoylation

**DOI:** 10.3389/fgene.2020.00174

**Published:** 2020-03-06

**Authors:** Natasha O. Saik, Nogi Park, Christopher Ptak, Neil Adames, John D. Aitchison, Richard W. Wozniak

**Affiliations:** ^1^Department of Cell Biology, University of Alberta, Edmonton, AB, Canada; ^2^Department of Basic Sciences, College of Veterinary Medicine, Mississippi State University, Starkville, MS, United States; ^3^New Culture, San Francisco, CA, United States; ^4^Seattle Children’s Research Institute, Seattle, WA, United States

**Keywords:** nuclear pore complex, gene positioning, gene expression, sumoylation, *INO1*, Ulp1, Siz2

## Abstract

In addition to their role in regulating transport across the nuclear envelope, increasing evidence suggests nuclear pore complexes (NPCs) function in regulating gene expression. For example, the induction of certain genes (e.g., yeast *INO1*) is accompanied by their movement from the nuclear interior to NPCs. As sumoylation has been linked to the regulation of chromatin spatial organization and transcriptional activity, we investigated the role of sumoylation in the expression and NPC recruitment of the *INO1* gene. We observed that induction of *INO1* is accompanied by both increased and decreased sumoylation of proteins associated with specific regions along the *INO1* locus. Furthermore, we show that the E3 ligase Siz2/Nfi1 is required for targeting the *INO1* locus to the NPC where it interacts with the SUMO isopeptidase Ulp1. Our data suggest that this interaction is required for both the association of *INO1* with the NPC and for its normal expression. These results imply that sumoylation is a key regulator of *INO1* targeting to the NPC, and a cycle of sumoylation and NPC-associated desumoylation events contribute to the regulation of *INO1* expression.

## Introduction

Small ubiquitin-related modifier (SUMO) is an ubiquitin-like peptide that is covalently attached to certain lysines in proteins. Sumoylation of proteins can result in changes to protein stability, subcellular localization, or interactions with other proteins. The vast majority of proteins modified by sumoylation are nuclear proteins and sumoylation and desumoylation events have been linked to the regulation of diverse group of nuclear processes including DNA replication, transcriptional control, and the spatial organization of the genome (Jentsch and Psakhye, 2013; Texari and Stutz, 2015; Zhao, 2018; Rosonina, 2019).

In *Saccharomyces cerevisiae*, the SUMO polypeptide is encoded by a single gene, *SMT3*. SUMO is translated with a C-terminal extension and later cleaved at a di-glycine motif by a SUMO peptidase to yield mature SUMO (Li and Hochstrasser, 1999). Like ubiquitination, SUMO conjugation is accomplished by a series of enzymes that first activate the mature peptide via ATP-dependent formation of a thioester bond (E1 enzyme, Aos1/Uba2 heterodimer in *S. cerevisiae*). Activated SUMO is subsequently handed-off to an active site cysteine in the E2 conjugating enzyme Ubc9. Ubc9 functions to directly transfer SUMO to a lysine in the target protein. In most cases, an E3 ligase aids SUMO-substrate specificity by mediating or stabilizing target interactions with the E2 (Geiss-Friedlander and Melchior, 2007). There are four known SUMO ligases in yeast, Zip3, Mms21, Siz1, and Siz2, the former functioning during meiosis while the latter three function in actively growing cells (Johnson, 2004; Jentsch and Psakhye, 2013). There is a significant overlap and redundancy in E3 ligase targets; however, E3 ligases have also been shown to have specific and independent functions (Makhnevych et al., 2007; Ferreira et al., 2011; Hannan et al., 2015). The SUMO conjugating system components are primarily present in the nucleoplasm (Srikumar et al., 2013) as are the vast majority of proteins modified by sumoylation (Panse et al., 2004; Wohlschlegel et al., 2004; Zhao et al., 2004; Zhou et al., 2004; Hannich et al., 2005; Wykoff and O’Shea, 2005).

Sumoylation is a reversible process. In budding yeast, there are two functionally distinct isopeptidases, Ulp1 and Ulp2. Both Ulp1 and Ulp2 deconjugate SUMO from target proteins; however, Ulp2 also suppresses poly-SUMO chain accumulation (Bylebyl et al., 2003), while Ulp1 carries out the essential role of processing pre-SUMO to mature SUMO (Li and Hochstrasser, 1999; Mossessova and Lima, 2000). *ulp1-ts* and *ulp2Δ* mutants exhibit very different overall sumoylation patterns (Li and Hochstrasser, 2000), and the desumoylation of specific sumoylated targets have been shown to be dependent on specific isopeptidases (Makhnevych et al., 2007; Felberbaum et al., 2012), indicating that each enzyme has distinct substrates. Ulp1 and Ulp2 also have distinct locations in the cell; Ulp2 is distributed throughout the nucleoplasm, while Ulp1 is associated with the nucleoplasmic face of nuclear pore complexes (NPCs) (Li and Hochstrasser, 2000; Panse et al., 2003).

The Ulp1 catalytic domain resides in its C-terminus, whereas several N-terminal domains of Ulp1 contribute to its association with NPCs (Li and Hochstrasser, 2003; Panse et al., 2003; Makhnevych et al., 2007). The N-terminal regions also bind to nuclear transport factors; residues 1–150 bind the import karyopherin Kap121, residues 150–340 bind the import karyopherin heterodimer Kap95/Kap60, and residues 340–403 contain a nuclear export signal that appears to interact with the export factor Xpo1 (Panse et al., 2003). Binding of Ulp1 to the NPC appears dependent on structures positioned on the nucleoplasmic face of the NPC, including proteins such as Mlp1, Mlp2, Nup60, and Nup2 (Zhao et al., 2004; Palancade et al., 2007; Srikumar et al., 2013). However, the molecular basis for these interactions has not been established.

The association of Ulp1 with NPCs is particularly intriguing and has led to the analysis of its role in various processes performed by NPCs, including the regulation of nuclear transport (Stade et al., 2002; Panse et al., 2006; Lewis et al., 2007), certain DNA repair pathways (Zhao et al., 2004; Palancade et al., 2007; Freudenreich and Su, 2016), and the regulation of gene expression (Texari et al., 2013; Bonnet et al., 2015; Abraham and Mishra, 2018). Among the various mechanisms by which NPCs can influence gene expression is their direct interactions with chromatin. NPCs interact with both transcriptionally repressed and active genes through interactions that appear to be mediated by transcription factors (TFs) of various types that bind specific chromatin sites and interact with different sets of Nups (Brickner et al., 2007; Ahmed et al., 2010; Light et al., 2010; Ruben et al., 2011; Van de Vosse et al., 2013; Brickner et al., 2019). These interactions influence both the spatial organization of associated genes and contribute to transcriptional state.

In yeast, numerous studies have examined the relocalization to NPCs of inducible genes following activation. Several genes have been shown to reside in the nuclear interior when repressed, but move to NPCs when induced. Well studied among these is the *INO1* locus (Brickner and Walter, 2004; Cabal et al., 2006; Brickner et al., 2007; Ahmed et al., 2010; Light et al., 2010). In the presence of inositol (repressive conditions), *INO1* is bound by the repressors Opi1 and Ume6, and the Rpd3(L) histone deacetylase complex, which repress *INO1* expression and association with the NPC. Following induction (inositol starvation), Opi1, presumably with Ume6 and Rpd3(L), dissociates from *INO1*. This is thought to be followed by the binding of TFs (Put3 and Cbf1) to cis-acting DNA elements (termed gene GRS1 and GRS2), which exhibit redundant functions in targeting *INO1* to an NPC (Loewen et al., 2003; Brickner and Walter, 2004; Shetty and Lopes, 2010; Brickner and Brickner, 2012; Randise-Hinchliff et al., 2016; Brickner et al., 2019). The SAGA complex (involved in transcription initiation) also appears to contribute to the association of active *INO1* with the NPC (Lo et al., 2001, 2005). Here, specific NPC components contribute to the efficient binding to *INO1* (Light et al., 2010). These interactions have been proposed to promote optimal transcription and mRNA export. However, our current lack of knowledge on the molecular basis for the interactions of *INO1*, and other active genes, with the NPC has limited our understanding of its significance.

Several Nups that play a role in the NPC-association of *INO1* also functionally interact with Ulp1 suggesting it is positioned at or near the site of gene association. Furthermore, alterations in sumoylation events have been shown to impact *INO1* transcription (Felberbaum et al., 2012). Consistent with this idea, Ulp1 has been previously shown to contribute to the activation and NPC-binding of the *GAL1* gene (Cabal et al., 2006; Texari et al., 2013). These observations and others implicating sumoylation in chromatin association with the NE and the regulation of gene expression has led us to investigate the role of sumoylation and desumoylation in the localization and expression of *INO1*. Our analysis of the roles of the SUMO ligase Siz2 and Ulp1 have revealed functions for sumoylation and desumoylation events in the NPC targeting and expression of activated *INO1*. We show that induction of *INO1* is accompanied by Siz2-dependent sumoylation of proteins associated with the *INO1* locus and propose that these modifications are required for targeting the gene to the NPC. Once at an NPC, Ulp1 interacts with sumoylated proteins associated with the induced *INO1* gene, primarily within its ORF. We propose that subsequent Ulp1-mediated desumoylation promotes expression and NPC association of activated *INO1*.

## Materials and Methods

### Media, Yeast Strains, and Plasmids

*Saccharomyces cerevisiae* strains used in this study were derived from YEF473A (Bi and Pringle, 1996) and are listed in Supplementary Table S1. Strains were grown in either fully supplemented synthetic media (SC media) (0.17% yeast nitrogen base, 0.5% ammonium sulfate, 2% glucose) or in synthetic media lacking inositol (INO^–^ media) or were grown in YPD (1% yeast extract, 2% bactopeptone, and 2% glucose). Plasmid bearing strains were grown in the appropriate synthetic dropout media (0.8% dropout powder, 0.17% yeast nitrogen base, 0.5% ammonium sulfate, 2% glucose) that either contained or lacked inositol.

Strain construction employed genome modifications performed using a one-step genomic integration method (Longtine et al., 1998), in which a DNA cassette was transformed into an appropriate strain using the lithium acetate/polyethylene glycol method (Gietz and Woods, 2002). DNA cassettes used to produce C-terminal Protein A, GFP, RFP, and mCherry gene fusions were made using a plasmid/PCR-based method (Longtine et al., 1998). DNA cassettes encoding the various *ulp1Δ* mutants were made by ligating together PCR generated DNA segments modified with specific restriction enzyme sites. For *ulp1Δ_1__–__150_* and *ulp1Δ_1__–__340_*, these DNA cassettes (bracketed) and restriction sites include: (*ULP1* 5′UTR)-*Eco*RI-(*ULP1* nucleotides from 451 within the ORF to 26 after the stop codon for *ulp1Δ_1__–__150_* and nucleotides from 1021 within the ORF to 26 after the stop codon for *ulp1Δ_1__–__340_*)-*Bam*HI-(marker gene: *NATMX* for *ulp1Δ_1__–__150_* and *KANMX* for *ulp1Δ_1__–__340_*)-*Spe*I-(*ULP1* 3′UTR). The *Eco*RI site introduces Glu-Phe codons after the Met start codon. The *ulp1Δ_150__–__340_* cassette consisted of (*ULP1* 5′UTR to *ULP1* nucleotide 450 within the ORF)-*Bss*HII-(*ULP1* nucleotides 1021–1861 within the ORF)-*Spe*I-(*ULP1* nucleotides 2–26 after the stop codon)-*Bam*HI-(marker gene *NATMX*)-*Spe*I-(*ULP1* 3′UTR). The *Bss*HII restriction site introduces Ala-Arg codons, while the *Spe*I restriction site at the 3′ end of *ulp1Δ_150__–__340_* overlaps with the stop codon and introduces a Lys to Asn codon substitution at the 3′ end of the ORF. PCR-based generation of DNA cassettes also introduced a point mutation in, *ulp1Δ_1__–__340_* resulting in an F610S amino acid residue substitution, and in *ulp1Δ_150__–__340_* resulting in an E409G residue substitution, that have no apparent effect on the relative function of these deletion derivatives. The *NUP53-ulp^340–621^* DNA cassette consisted of (*ULP1* 5′UTR)-*Nde*I-(*NUP53*)-*Sal*I-(*ULP1* nucleotides from 1018 within the ORF to 26 after the stop codon)-*Spe*I-(*ULP1* nucleotides 2–26 after the stop codon)-*Bam*HI-(marker gene *NATMX*)-*Spe*I-(*ULP1* 3′UTR). The *Sal*I restriction site adds Val-Asp codons at the fusion point between *NUP53* and *ulp1^340–621^*. The *Spe*I restriction site at the 3′ end of *ulp^340–621^* overlaps with the stop codon and introduces a Lys to Asn codon substitution at the 3′ end of the ORF. PCR-based generation of *NUP53-ulp^340–621^* introduced point mutations resulting in an E409G, and V584A amino acid substitutions in *ulp^340–621^* that have no apparent effect on the relative function of this fusion.

To visualize *INO1* gene localization, a previously described genomic tagging system was employed (Straight et al., 1996). *GFP-lacI-HIS* was integrated at the *his-Δ200* locus using the pAFS78 plasmid (Robinett et al., 1996). To tag the *INO1* locus, the plasmid pAFS52.*INO1* was made by cloning PCR amplified *INO1*, containing *Xho*I sites at its 5′ and 3′ ends, into pAFS52 (Straight et al., 1996). *Bgl*II digested pAFS52.*INO1* was then transformed into yeast to integrate the *lacO_256_-TRP* array. This resulted in a duplication of the *INO1* locus with *lacO_256_-TRP* found between the duplicates.

Plasmids used here are derivatives of pRS315 (pEMPTY) (Sikorski and Hieter, 1989) and include pRS315.Ulp1-GFP and pRS315.ulp1^*CSDN*^-GFP (Elmore et al., 2011), as well as pRS315.Ulp1 and pRS315.ulp1^*CSDN*^ (this work).

### *INO1* Gene Induction

To induce *INO1* gene expression, cell cultures were grown overnight at room temperature in SC media, diluted into fresh SC media to an OD_600_ = 0.2, and then grown at 30°C until the cultures reached mid-log phase (OD_600_ = ∼0.8). A sample of these cultures was then taken as the uninduced control, and processed as required. A second sample of cells from these cultures were collected by centrifugation, washed once with water, and then resuspended in INO^–^ media to an OD_600_ = 0.5 to induce INO1 expression. These cultures were then grown at 30°C. Cells were collected at the stated time points and processed as indicated.

### qRT-PCR for *INO1* Gene Expression

*INO1* induction was carried out as described above and, at each time point, an OD_600_ = 10 equivalent of cells was pelleted and processed. RNA preparation from these cells and subsequent real-time qRT-PCRs were performed as previously described (Wan et al., 2009). cDNA was amplified using 2 μg of DNAse-treated RNA that was reverse transcribed using 200 units of Superscript II reverse transcriptase (Invitrogen) at 42°C for 50 min and the resulting cDNAs were diluted 100-fold. Reactions were assembled using SYBR green super mix (Quanta), as per the manufacturer’s protocol, and included sense (S) and antisense (AS) primers against *ACT1* (S-GGATTCCGGTGATG GTGTTA, AS-TCAAATCTCTACCGGCCAAA) and *INO1* (S-CACCAT GGAAAACCTCTTGC, AS-GGGGACACCTTCCAAGATAGA) as previously described (Brickner et al., 2007). Reactions were carried out on an Mx3000P QPCR System (Agilent Technologies). *INO1* mRNA levels were normalized relative to *ACT1* mRNA levels from three independent qRT-PCR analyses.

### ChIP for Protein Localization at *INO1*

*INO1* induction was carried out as described above and, at each time point, an OD_600_ = 50 equivalent of cells was pelleted and processed. Chromatin immunoprecipitation experiments were performed as previously described (Wan et al., 2009). For immunoprecipitation, 4 μl of rabbit polyclonal anti-PrA (Sigma) antibody or 4 μl of rabbit polyclonal anti-Smt3 (SUMO) antibody (Wozniak lab) was prebound to 100 μl of Protein G Dynabeads (Invitrogen). Immunoprecipitated DNA was recovered and analyzed by qRT-PCR as described above. Sense (S) and antisense (AS) primers used for qRT-PCR included: Chromosome V intergenic region (S-ACATTCTTGGAAA CCCATCG, AS-TCGTATCATGATTTAGCGTCGT); *INO1* regions: *GRS1* (S-TC GTTCCTTTTGTTC TTCACG, AS-GCCTCCGCATATTTCA CATT), *A* (S-AAATGCGGCATGTGAAAAGT, AS-AGAG GTG CGCTTTCTCTGC), *B* (S-AGAGAAAGCGCACCTCTGC, AS- (AGGAACCCGACAACAGAACA), *C* (S-CGACAAGTGCACG TACAAGG, AS-CAGTGGGCGTTACATCGAA), *D* (S-CTTC GGCTCC ATGACTCAAT, AS-GCTAACCATGGGCAACAG AG), *E* (S-GGACTCAAAAGTGGCAATGG, AS-TCAAGGGC GTAGCCAGTAAA), *F* (S-CGTCTTAAAAGGGGCGTTTT, AS-TTTACTGAGG TGGCCCTTGA). To quantify the ChIP experiments, we first expressed the amount of *INO1* or Chromosome V intergenic region sequence immunoprecipitated as a percentage of total input (% of input). Using these values, we calculated ratios comparing the% of input from each region of the *INO1* gene to the% of input for the Chromosome V intergenic region for both uninduced (cells grown in the presence of inositol) and induced (cells grown in the absence of inositol for 1 and 3 h) samples. Relative fold change for the induced samples was then calculated by dividing the induced ratio determined for a given region of the *INO1* gene by the uninduced ratio for that same region.

### Fluorescence Microscopy

To image the GFP-lacI/lacO_256_ tagged *INO1* locus, cell cultures were treated as described above for *INO1* gene induction. Cells from 1 ml of culture were pelleted by centrifugation, washed once with the appropriate synthetic media, and then resuspended in the same media; 1.5 μl of cells was then spotted onto a microscope slide for live-cell image acquisition. Epifluorescence images were acquired on a DeltaVision Elite imaging system (GE Healthcare Life Sciences) at 60x magnification using a 1.42 NA oil, Plan Apo N objective (Olympus). Images were collected and saved as 15 × 0.2 μm z-stacks using SoftWoRx software (version 6.5.2, GE Healthcare Life Sciences), then rendered and analyzed using Image J (NIH). *INO1* localization (GFP-lacI signal) was assessed relative the nuclear periphery (Nup49-mRFP signal) and was considered to colocalize with NPCs when the GFP-lacI focus fully or partially overlapped with Nup49-mRFP, similar to the previously described method (Brickner and Walter, 2004; Brickner and Brickner, 2010).

To assess the localization of the various Ulp1-GFP and Ulp1-mCherrry derivatives, strains producing these derivatives were grown in YPD media at 30^*o*^C to mid-log phase; 1 ml of cells from each culture was then pelleted by centrifugation, washed once with 1 ml of SC media, and resuspended in 20 μl of SC media; 1.5 μl was then spotted onto a microscope slide for epifluorescence imaging. Images were acquired using an Axio Observer.Z1 microscope (Carl Zeiss, Inc.), equipped with an UPlanS-Apochromat 100x/1.40 NA oil objective lens (Carl Zeiss, Inc.) and an AxioCam MRm digital camera with a charge-coupled device (Carl Zeiss, Inc.). Images were acquired in a single focal plane through the center of nuclei. Images were saved using AxioVision (Carl Zeiss, Inc.) software and rendered for display using Image J (NIH) software.

### Western Blot

Ulp1-GFP and various ulp1Δ-GFP derivatives were detected using western blot. Proteins from cells lysates were separated by SDS-PAGE and then transferred to nitrocellulose membranes. Membranes were incubated in blocking buffer (TBS containing 0.1% Tween-20 and 5% milk powder) for at least 1 h at room temperature. Blocking buffer was then removed and replaced with fresh blocking buffer supplemented with rabbit polyclonal antibodies directed against GFP, GSP1, or SUMO (Makhnevych et al., 2007) then incubated overnight at 4°C. Membranes were then washed three times using 0.1% Tween-20 in TBS, followed by incubation in blocking buffer supplemented with goat anti-rabbit HRP conjugated antibody (BioRad) at a 1:10,000 dilution for at least 1 h at room temperature. Membranes were then washed three times using 0.1% Tween-20 in TBS. Bound anti-rabbit HRP conjugated antibody was detected by chemiluminescence (Amersham) using an ImageQuant LAS 4000 (GE) imaging system.

## Results

### Activation of the *INO1* Gene Is Accompanied by Sumoylation of Associated Proteins

**FIGURE 1:**
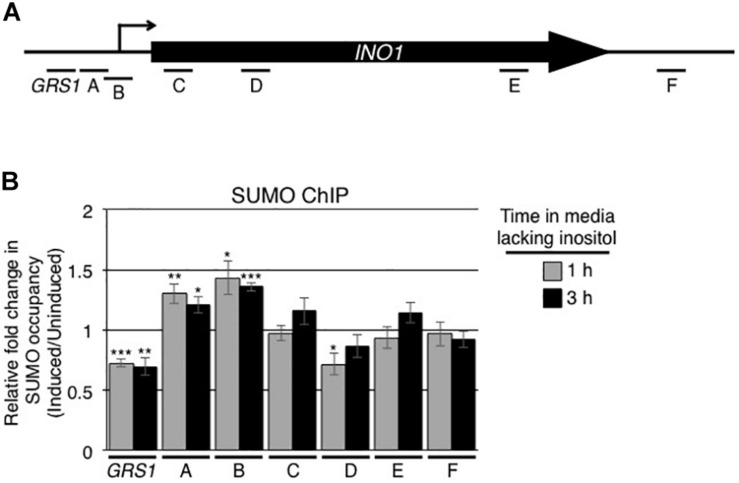
Induction of *INO1* leads to changes in the sumoylation of *INO1*-associated proteins. **(A)** Diagram of the *INO1* locus showing the relative positions of the transcriptional start site (arrow), the *GRS1* sequence, the open reading frame, and regions targeted for the ChIP analysis shown in **B**. **(B)** WT cells were grown in medium containing inositol (repressing/uninduced conditions) to an OD_600_ of ∼0.8, washed, and transferred to medium lacking inositol (inducing conditions). Cells were then subjected to ChIP analysis using antibodies directed against the SUMO polypeptide (anti-SUMO antibody) prior to and at 1 and 3 h post induction of *INO1*. qRT-PCR was used to quantify levels of DNA corresponding to the various regions of *INO1* (see **A**) bound to sumoylated proteins. Shown is the relative fold change in the occupancy of sumoylated proteins associated with the various regions of *INO1* at the indicated times after induction relative to the uninduced samples (see section “Materials and Methods”). Results are the means ± SEM of five biological replicates. Asterisks indicate a significant difference relative to uninduced as determined by a Student’s paired *t*-test. *p < 0.05, **p < 0.01, ***p < 0.001.

Following their activation, numerous yeast genes are repositioned from the nuclear interior to the nuclear envelope (Brickner and Walter, 2004; Texari et al., 2013; Brickner et al., 2019). A well-studied example is the *INO1* gene. When cells are switched from medium containing inositol to medium lacking this carbon source, the *INO1* gene is induced and the gene locus is targeted to NPCs (Brickner and Walter, 2004; Cabal et al., 2006; Brickner et al., 2007; Ahmed et al., 2010; Light et al., 2010). Since changes in the expression of genes are often accompanied by changes in the levels of sumoylation of associated TFs and other chromatin-associated proteins, we examined whether the induction of *INO1* alters the sumoylation state of proteins associated with the *INO1* locus. To test this, antibodies directed against SUMO were used in ChIP analysis targeting the *INO1* gene prior to and following induction. For these experiments, various sets of oligonucleotides were used to detect interacting regions along the *INO1* gene (Figure 1A). Prior to induction, the sumoylation state of chromatin associated proteins within the *GRS1* and ORF regions of the *INO1* gene were higher than that detected in a control intergenic region (Supplementary Figure S1B). Following induction, we observed significant changes in the levels of sumoylated proteins associated with specific regions of the gene (Figure 1B). In a 5′ region containing the previously identified *INO1* gene recruitment sequence 1 (GRS I), we observed a decrease in sumoylation of associated proteins while adjacent regions containing the transcriptional start site showed increases. Downstream regions within the ORF and the 3′ regions showed little or no change in the levels of associated sumoylated proteins.

### The SUMO Ligase Siz2 Is Required for Recruitment of the *INO1* Locus to the NE

**FIGURE 2:**
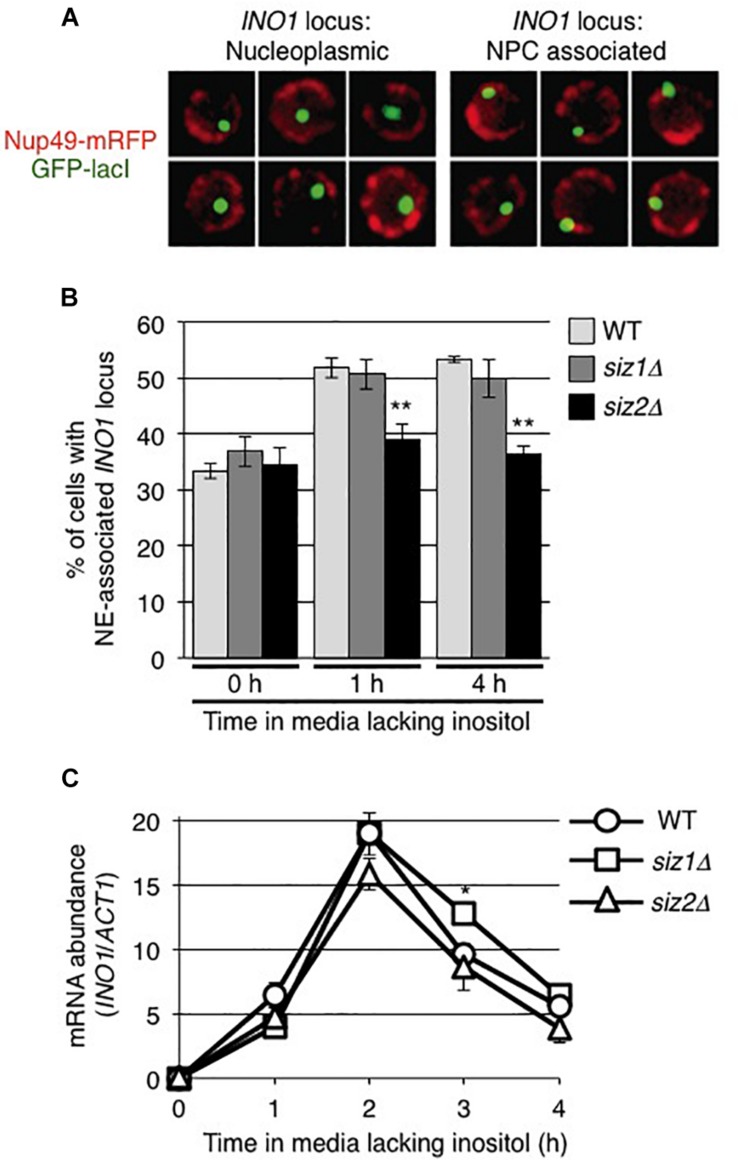
The SUMO ligase Siz2 is required for NE-association, but not transcription, of induced *INO1*. Localization of the *INO1* locus prior to and following *INO1* induction was examined in cells containing the *INO1-lacO_256_* construct and producing GFP-lacI (green). The position of the nuclear envelope (NE) in these cells relative to the *INO1-lacO* locus was determined using Nup49-RFP (red). **(A)** Examples of cell nuclei in which the *INO1* locus (green) was (left panels) or was not (right panels) associated with the nuclear periphery (red) are shown. *INO1* was scored to be NE-associated if the GFP-lacI signal fully or partially overlapped with the Nup49-mRFP signal. **(B)** The percentage of cells showing NE-association of *INO1-lacO* prior to (0 h) or at 1 and 4 h after induction was determined in the indicated strains. Results are the means ± SD of three biological replicates. At least 100 cells were counted for each sample. **(C)** Levels of *INO1* mRNA were examined in the indicated strains. Cell cultures were grown in medium containing inositol (repressing conditions) to an OD_600_ of ∼0.8 and then washed and placed in medium lacking inositol (inducing conditions) for the indicated times. Levels of mRNA encoded by the *INO1* gene were evaluated by qRT-PCR and their abundance relative to *ACT1* mRNA was determined. Results are the means ± SEM of three biological replicates. Asterisks indicate a significant difference from WT as indicated by Student’s unpaired *t*-test, *p < 0.05, **p < 0.01.

Our observations that the levels of sumoylated proteins bound to the *INO1* locus, in particular those associated with 5’ regions containing the GRS1 sequence, changed upon induction led us to investigate the role of sumoylation in the NPC association of *INO1*. As the SUMO ligase Siz2 had been previously shown to play a role in the nuclear envelope association of telomeres (Ferreira et al., 2011; Churikov et al., 2016; Lapetina et al., 2017), we examined the role of Siz2, and the related SUMO ligase Siz1, in *INO1* localization following its induction. The position of *INO1* was monitored by tagging with an adjacent *lacO*_256_ cassette in cells producing the GFP-lacI protein (Brickner and Walter, 2004; Ahmed et al., 2010; see Figure 2A). Induction of *INO1* led to a rapid (within 1 h) accumulation of *INO1:lacO_256_*/GFP-lacI foci at the nuclear periphery in WT cells and those lacking Siz1 (*siz1Δ*) (Figure 2B). By contrast, we observed that in cells lacking Siz2 (*siz2Δ*), *INO1* recruitment to the NE was not observed following induction. We also measured *INO1* mRNA levels following induction in these various strains, and observed no differences in the induction profiles (Figure 2C). These results suggest that Siz2 is required for *INO1* binding to the nuclear periphery upon induction, but its loss has no significant effect on *INO1* expression.

The requirement of Siz2 for the binding of induced *INO1* to the NE led us to examine whether Siz2 contributed to sumoylation at the *INO1* locus. To test this idea, we examined levels of sumoylated proteins at the *INO1* locus in the *siz2Δ* mutant. In contrast to WT cells, the *siz2Δ* mutant cells showed reduced levels of sumoylation within the ORF of the *INO1* locus in uninduced cells. Moreover, induction of *INO1* did not significantly alter sumoylated proteins levels along the INO1 locus (Supplementary Figure S1C). These results are consistent with Siz2 functioning in the sumoylation of proteins associated with regions of the *INO1* locus prior to and following *INO1* activation.

**FIGURE 3:**
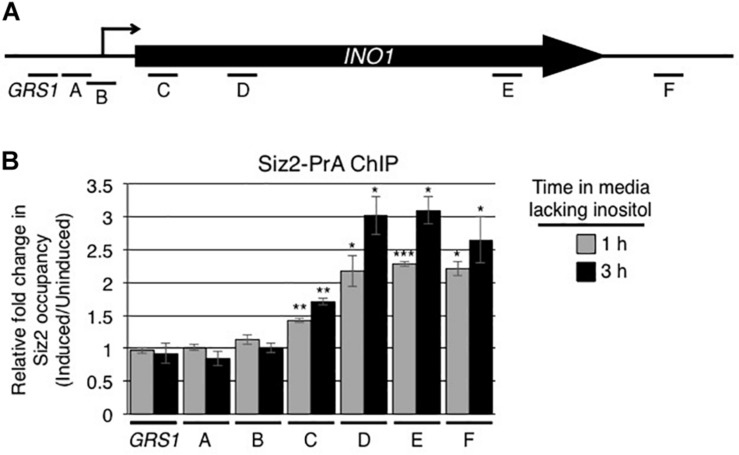
Induction of *INO1* is accompanied by recruitment of Siz2 to the *INO1* locus. **(A)** Diagram of the *INO1* locus and regions detected by ChIP analysis (see Figure 1A) in **B**. **(B)** Cells expressing protein A tagged Siz2 (Siz2-PrA) were grown as described in the Figure 1 legend, and harvested at 0, 1, and 3 h after *INO1* induction. Changes in Siz2-PrA occupancy along the *INO1* locus following induction were examined by ChIP and qRT-PCR using primer pairs that amplify regions of *INO1* indicated in **A**. Relative fold change in Siz2-PrA binding was determined as described for SUMO binding in Figure 1. Results are the means ± SEM of at least three biological replicates. Asterisks indicate a significant difference relative to uninduced as determined by a Student’s paired *t*-test. *p < 0.05, **p < 0.01, ***p < 0.001.

We also examined whether Siz2 physically interacted with the *INO1* locus upon activation of the *INO1* gene. Using protein A tagged Siz2 (Siz2-PrA) and ChIP analysis, we examined the binding of Siz2-PrA along the *INO1* locus prior to and following 1 and 3 h induction (Supplementary Figure S2 and Figure 3B). In uninduced cells, we detected significantly higher levels of Siz2-PrA bound to the *GRS1*-containing region relative to the intergenic control, while other regions show no enrichment (Supplementary Figure S2). Upon induction of *INO1*, we observed no significant change in Siz2-PrA binding within 5′ regions of *INO1* locus (from site GRS I to site B, see Figure 3A for map). However, Siz2-PrA occupancy within the *INO1* ORF (region C to F) increased markedly upon induction (Figure 3B). These results are consistent with Siz2 functioning in the sumoylation of proteins associated with various regions of the *INO1* locus both prior to (upstream of the ORF) or in response to *INO1* activation (within the ORF).

### *INO1* Interacts With Ulp1 Following Induction

**FIGURE 4:**
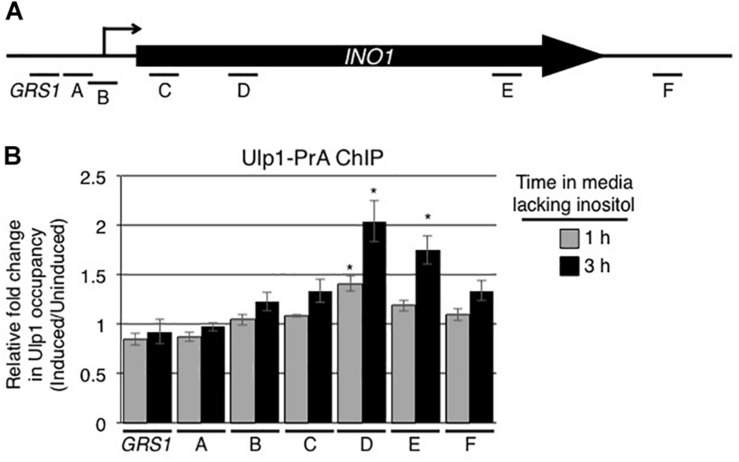
Ulp1 interacts with the induced *INO1* gene. **(A)** Diagram of the *INO1* locus and regions detected by ChIP analysis (see Figure 1A). **(B)** Cells producing protein A tagged Ulp1 (Ulp1-PrA) were grown as described in the Figure 1 legend, and harvested at 0, 1, and 3 h after *INO1* induction. Changes in Ulp1-PrA occupancy throughout the *INO1* locus upon induction were examined by ChIP and qRT-PCR using primer pairs that amplify regions of *INO1* indicated in **A**. Relative fold change in Ulp1-PrA binding was determined as described for SUMO binding in Figure 1. Results are the means ± SEM of three biological replicates. Asterisks indicate a significant difference relative to uninduced as determined by a Student’s paired *t*-test. *p < 0.05.

The association of activated genes with NPCs is thought to occur through interactions between the transcriptional machinery and proteins located on the nuclear face of the NPC. Among these, Nup60 and the related proteins Mlp1/Mlp2 are required for *INO1* association with NPCs (Brickner et al., 2007; Ahmed et al., 2010; Light et al., 2010; Brickner et al., 2019). These NPC proteins are also required for the association of the desumoylase Ulp1 with NPCs (Zhao et al., 2004; Palancade et al., 2007; Srikumar et al., 2013). As Ulp1 is an important regulator of protein sumoylation, we investigated its role in the expression and NPC targeting of the induced *INO1* gene. We tested whether the *INO1* gene physically interacts with Ulp1. Using ChIP, no significant enrichment of Ulp1-pA was detected along the *INO1* locus prior to induction (Supplementary Figure S2C). However, following activation, we observed that Ulp1 occupancy significantly increased specifically within the *INO1* ORF (Figure 4). By contrast, no detectable change was observed in regions upstream of the ORF. These results suggest that induction of *INO1* is followed by the association of Ulp1 with specific regions of the *INO1* gene.

### NPC Recruitment and Expression of *INO1* Require NPC-Associated Ulp1

To evaluate the role of Ulp1 in the recruitment of activated *INO1* to NPCs, we examined whether *ulp1* mutants that lacked domains required for Ulp1 association with NPCs altered the localization of the *INO1* gene. Our strategy was to uncouple Ulp1 from NPCs without altering its catalytic domain (contained within amino-acid residues 403-621) and its essential function in SUMO maturation (Li and Hochstrasser, 2003). Mutants lacking either of the two of previously described NPC binding domains of Ulp1, residues 1–150 (ulp1Δ_1__–__150_) or 150–340 (ulp1Δ_150__–__340_), were previously shown to still bind to NPCs (Li and Hochstrasser, 2003; Panse et al., 2003). However, a mutant lacking both domains, i.e., residues 1–340 (ulp1Δ_1__–__340_), showed greatly reduced levels of NPC association (Panse et al., 2003). Each of these truncation mutations was integrated within the context of the endogenous *ULP1* locus by replacing the endogenous ORF and thus retaining the endogenous promoter and single copy number of the gene. This approach reduces the potential for artifacts arising from elevated levels of Ulp1 derived from plasmid-encoded genes. An examination of the integrated GFP-tagged versions of these mutants revealed similar protein levels to WT (Supplementary Figure S3B) and a localization pattern consistent with previous reports, with both the ulp1Δ_1__–__150_-GFP and the ulp1Δ_150__–__340_-GFP showing NE levels similar to WT Ulp1, while ulp1Δ_1__–__340_-GFP showed low levels of NE-association with a concomitant increase in cytoplasmic and nucleoplasmic localization relative to WT Ulp1-GFP (Supplementary Figure S3A). Similarly, sumoylation patterns in the ulp1Δ_1__–__150_ and the ulp1Δ_150__–__340_ mutants appeared largely similar to WT cells. However, levels of sumoylated proteins are slightly reduced in the ulp1Δ_1__–__340_ mutant (Supplementary Figure S3C).

**FIGURE 5:**
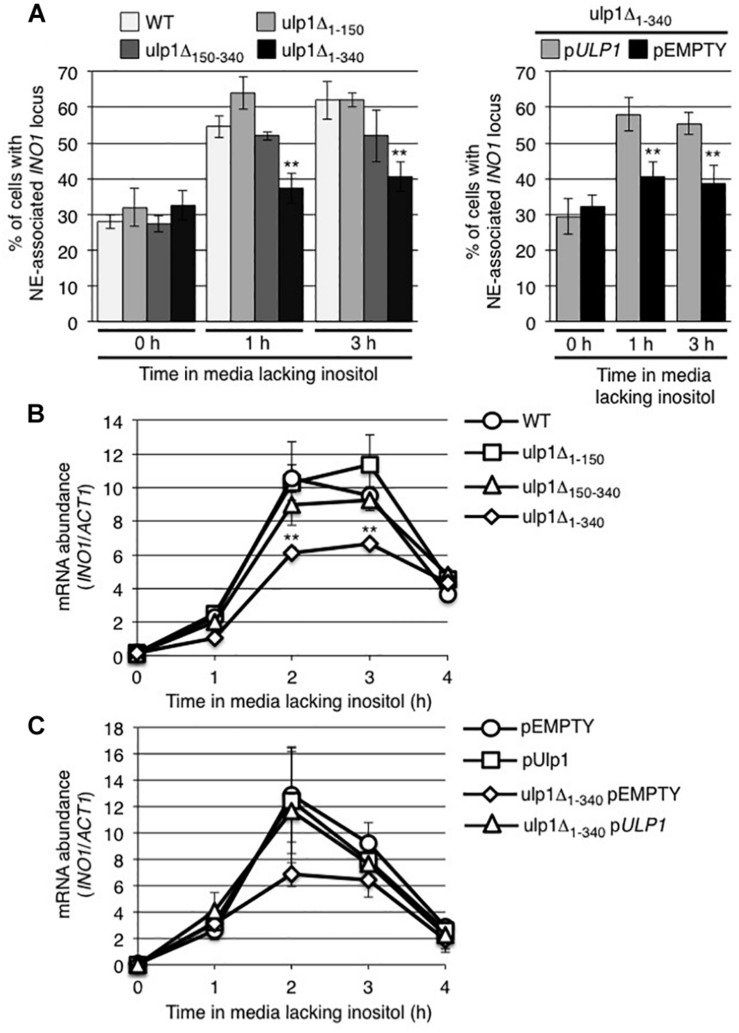
Ulp1 is required for *INO1* expression and NE-association following induction. **(A)** The percentage of cells showing NE-association of *INO1-lacO_256_* was determined as described in Figure 2 prior to (0 h) and at 1 and 3 h post induction in WT and the indicated *ulp1* mutant strain backgrounds. Shown on the right are the results of similar experiments performed on a *ulp1*Δ*_1__–__340_* strain transformed with either an empty plasmid (pEMPTY) or a plasmid containing a version of WT *ULP1* (p*ULP1*). Results are the means ± SD of three or more biological replicates. At least 50 cells were counted for each sample. **(B,C)** Levels of mRNA encoded by the *INO1* gene were evaluated by qRT-PCR as described in Figure 2 following induction for the specified times in the indicated strains. Results are the means ± SEM of three biological replicates. Note, **C** shows data from the indicated strains transformed with either an empty plasmid (pEMPTY) or a plasmid containing a version of WT *ULP1* (p*ULP1*). Asterisks indicate a significant difference from WT as indicated by a Student’s unpaired *t*-test, **p < 0.01.

We examined the effects of *ulp1* truncation mutants on the inducible recruitment of *INO1* to NPCs (Figure 5A). Induction of *INO1* led to a rapid (within 1 h) accumulation of *INO1* foci at the nuclear periphery in cells producing the ulp1Δ_1__–__150_ or ulp1Δ_150__–__340_ truncation, similar to that observed in WT cells. By contrast, cells producing the ulp1Δ_1__–__340_ mutant showed no localization of *INO1* to the NE following induction. Furthermore, in monitoring mRNA levels at various times post induction, we found that *INO1* transcript levels were reduced in the *ulp1Δ_1__–__340_* mutant and failed to reach levels detected in the *ulp1Δ_1__–__150_*, *ulp1Δ_150__–__340_*, or WT strains (Figure 5B). Importantly, both the recruitment of *INO1* to the NE and WT levels of *INO1* mRNA levels could be restored in the *ulp1Δ_1__–__340_* mutant by the introduction of WT *ULP1* (Figures 5A,C), suggesting the phenotype detected in *ulp1Δ_1__–__340_* mutant arises from the loss of Ulp1 at NPCs, and not the presence of the ulp1Δ_1__–__340_ mutant protein outside of the NPC.

**FIGURE 6:**
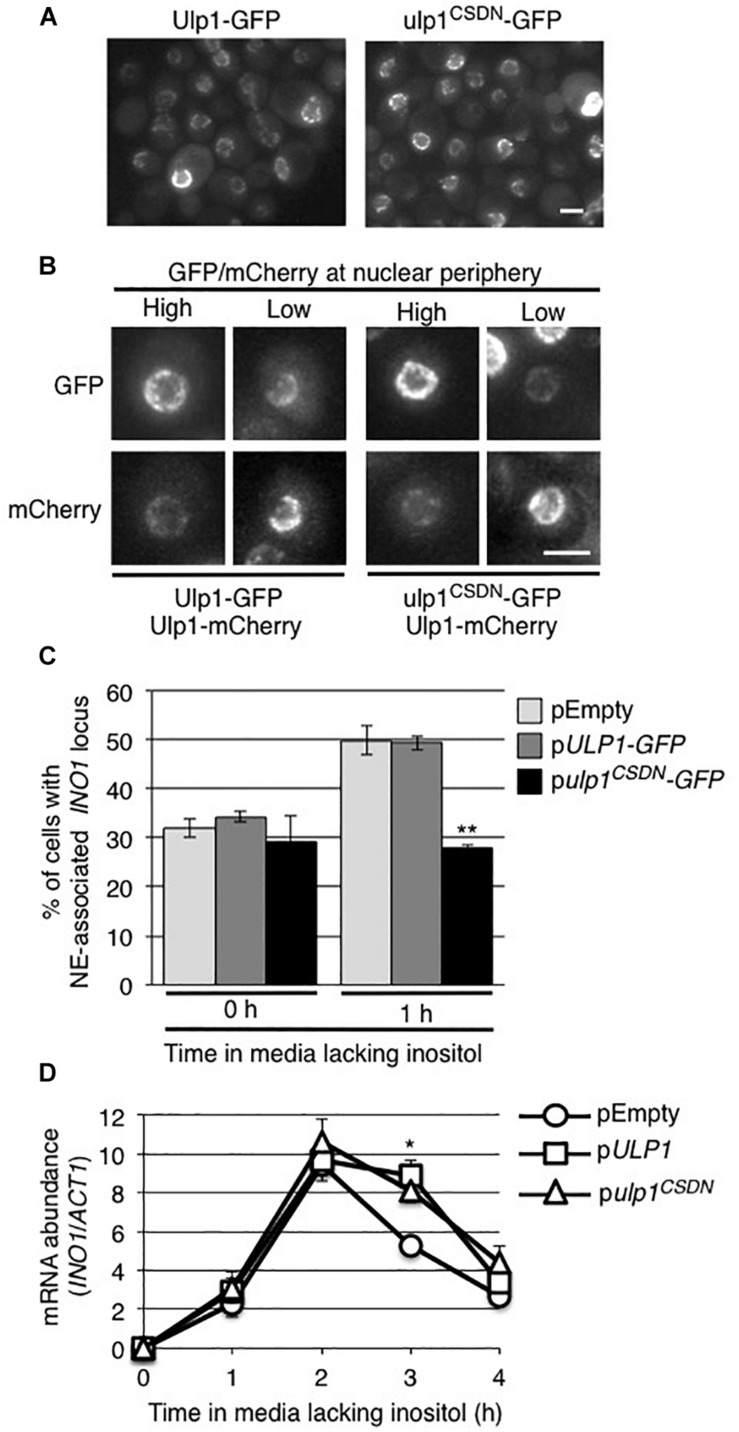
The *ulp1-CSDN* mutant inhibits *INO1* targeting to the NE. **(A)** Shown are images of WT cells producing exogenously expressed, plasmid-encoded Ulp1-GFP or Ulp1^*CSDN*^-GFP. **(B)** The localization of plasmid-encoded Ulp1-GFP or Ulp1^*CSDN*^-GFP in cells producing mCherry-tagged endogenous wild-type Ulp1 (Ulp1-mCherry) was examined. Shown are cells containing relatively high or low levels of the GFP fusion, likely stemming from cell to cell variability in plasmid copy number. Competition of NE-associated binding between the plasmid-encoded Ulp1-GFP or Ulp1^*CSDN*^-GFP and endogenous Ulp1-mCherry proteins is indicated by the relative GFP and mCherry signal intensities at the NE. Scale bars = 2 μm. **(C)** Recruitment of *INO1* to the NPCs was measured as in Figure 2 in a WT strain containing *INO1-lacO_256_*/GFP-lacI and the indicated plasmid. Results are the means ± SD of three or more biological replicates. At least 50 cells were counted for each sample. **(D)** Levels of mRNA encoded by the *INO1* gene were evaluated by qRT-PCR as described in Figure 2 following induction for the specified times in the indicated strains. Asterisks indicate a significant difference from WT (pEMPTY) as indicated by a Student’s unpaired *t*-test, *p < 0.05, **p < 0.01.

The effects of the *ulp1Δ_1__–__340_* mutant on the *INO1* localization and expression could occur as a consequence of the loss of functions linked to its N-terminal domain (residues 1–340) or the loss of Ulp1 isopeptidase activity at the NPC. To investigate these possibilities, we examined the effects of expressing a *ulp1* double point mutant (*ulp1*^*CSDN*^) that abrogates SUMO binding and isopeptidase activity (Mossessova and Lima, 2000; Elmore et al., 2011) but does not alter its N-terminal domain or targeting to NPCs. This catalytically dead *ulp1*^*CSDN*^ does not support cell viability in the absence of WT Ulp1 (Elmore et al., 2011), thus we expressed the *ulp1*^*CSDN*^ mutant in WT cells and assessed whether the mutant exhibited dominant negative phenotypes. As shown in Figure 6A, ulp1^*CSDN*^-GFP localizes to NPCs, consistent with the known functionality of its N-terminus. Levels of the mutant protein at the nuclear periphery varied between cells, likely due to cell-to-cell variations in the amount of ulp1^*CSDN*^-GFP (arising from cell-to-cell variation in the plasmid encoded gene). Inspection of these cells revealed that the amount of ulp1^*CSDN*^-GFP at the NE appeared inversely proportional to the amount of endogenous WT Ulp1p at the same locale, suggesting the mutant protein was capable of competing with the WT protein for NPC binding sites (Figure 6B).

The expression of the *ulp1*^*CSDN*^ mutant had little effect on cell growth and no striking changes were seen in global protein sumoylation patterns (Supplementary Figures S4A,B). However, when we examined the localization of *INO1* following induction, the presence of the ulp1^*CSDN*^ mutant protein inhibited the *INO1* locus from relocating to the NE (Figure 6C). By contrast, plasmid-borne *ULP1* did not alter induction-dependent *INO1* association with the NE. These results led us to conclude that the association of Ulp1 catalytic activity at the NPC is required for recruitment of the *INO1* locus. However, cells producing the ulp1^*CSDN*^-GFP mutant did not exhibit altered *INO1* expression following induction (Figure 6D), suggesting that the mutant may not exhibit a dominant negative phenotype with respect to *INO1* expression.

**FIGURE 7:**
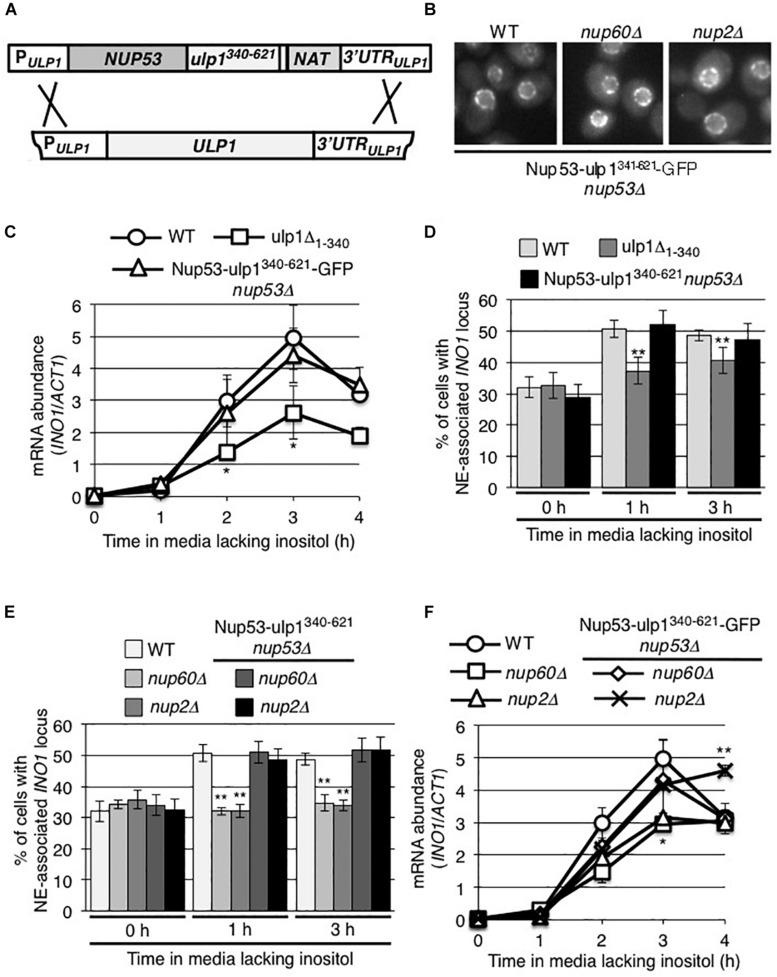
The NPC association of the C-terminal domain of Ulp1 is sufficient to support recruitment of induced *INO1*. **(A)** Endogenous *ULP1* was replaced by a *NUP53-ulp1^340– 621^* chimera under the control of the *ULP1* promoter in a haploid yeast strain. A schematic representation of the construction of this chimeric gene is shown. **(B)** Localization of the Nup53-ulp1*^340– 621^* fusion protein C-terminally tagged with GFP was examined in an otherwise WT background or in strains lacking *NUP60* or *NUP2*. **(C,F)** Levels of mRNA encoded by the *INO1* gene were evaluated by qRT-PCR following induction for the indicated times; **(C)** in WT, *NUP53-ulp1^340– 621^*, and *ulp1Δ_1__–__340_* strains and **(F)** in WT, *nup60Δ*, *nup2Δ*, *NUP53-ulp1^340– 621^ nup60Δ, and NUP53-ulp1^340– 621^ nup2Δ* strains as described in Figure 2. Results are the means ± SEM of three biological replicates. **(D,E)** Localization of the *INO1-lacO_256_* locus was examined prior to (0 h) or at 1 and 3 h after induction in the indicated strain backgrounds. Note that the data shown here for *ulp1Δ_1__–__340_* in **D** are the same as that in Figure 5A and are shown here for comparison. Localization of *INO1-lacO_256_* locus was assessed as described in Figure 2. Results are the means ± SD of at least three biological replicates. For **D** and **E**, at least 100 cells were counted for each replicate. Asterisks indicate a significant difference from WT samples at the corresponding time points as indicated by Student’s unpaired *t*-test. *p < 0.05, **p < 0.01.

Our analysis of the *ulp1*^*CSDN*^ mutant suggests a role for the Ulp1 catalytic activity in the association of induced *INO1* with NPCs; however, these data provided no insight into its function in *INO1* expression. Therefore, we asked whether positioning of the Ulp1 catalytic domain at NPCs would be sufficient for *INO1* expression. To do this, we constructed a chimeric gene encoding the Ulp1 catalytic domain (residues 340–621) fused to the C-terminus of the nucleoporin Nup53 (Figure 7A). The Nup53-ulp1^340–621^ fusion protein showed a similar localization pattern to that observed for nucleoporins, consistent with its association with NPCs (Figure 7B). Moreover, cells producing the Nup53-ulp1^340–621^ protein, and lacking endogenous Ulp1, grew similar to WT cells (Supplementary Figure S4C), suggesting that the Ulp1 catalytic domain of the fusion protein could replace the essential function of WT Ulp1. In these Nup53-ulp1^340–621^ producing cells, we observed levels of *INO1* mRNA production following induction similar to that detected in WT cells, and analysis of *INO1* localization showed its recruitment to the NE was comparable to that seen in WT cells (Figures 7C,D). Thus, positioning of the Ulp1 catalytic domain at NPCs was sufficient to support *INO1* expression and NPC association.

Several *nup* mutants have been previously shown to inhibit post-induction *INO1* association with the NE, including a strain lacking Nup60 (*nup60Δ*) or Nup2 (*nup2Δ*) (Ahmed et al., 2010; Light et al., 2010; also see Figure 7E). Both Nup60 and Nup2 are functionally linked to Ulp1; the loss of Nup60 results in decreased cellular levels of Ulp1 (Palancade et al., 2007) and Nup2 has been reported as a SUMO target and a Ulp1 interacting partner (Hannich et al., 2005; Srikumar et al., 2013; Folz et al., 2019). Therefore, we tested whether the Nup53-ulp1^340–621^ protein could rescue the *INO1* targeting defects in the *nup60Δ* and *nup2Δ* mutants. In these mutants, the Nup53-ulp1^340–621^-GFP fusion was visible at the NE in a characteristic NPC pattern (Figure 7B). Importantly, we observed that production of the Nup53-ulp1^340–621^ protein in the *nup60Δ* and *nup2Δ* mutants rescued inducible *INO1* recruitment to the NPC (Figure 7E). Moreover, an examination of the expression of the *INO1* gene in these strains revealed that they produced WT levels of *INO1* mRNA (Figure 7F). On the basis of these data, we conclude that the defects previously detected in the *nup60Δ* and *nup2Δ* mutants are functionally linked to Ulp1.

### NPC-Associated Ulp1 Regulates Sumoylation Levels of Proteins Associated With the *INO1* ORF

**FIGURE 8:**
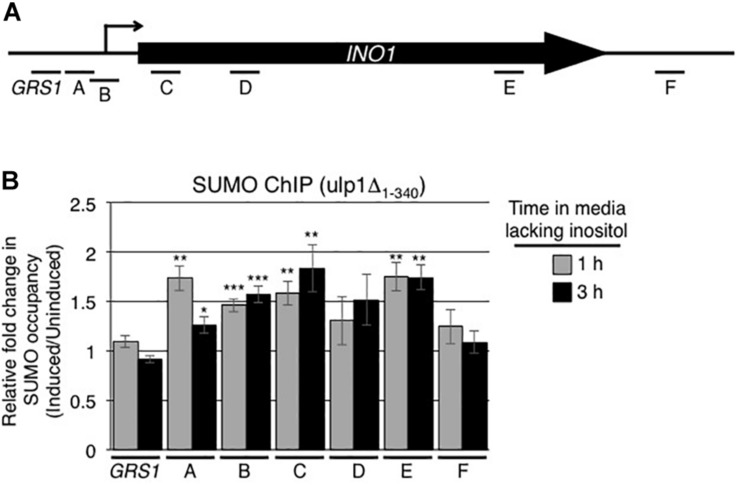
Ulp1 regulates sumoylation levels of proteins associated with the *INO1* ORF. **(A)** Diagram of the *INO1* locus and regions detected by ChIP analysis (see Figure 1A). **(B)** *ulpΔ_1__–__340_* cells were subjected to ChIP analysis using antibodies directed against the SUMO polypeptide (anti-SUMO antibody) prior to and at 1 and 3 h post induction of *INO1*. qRT-PCR was used to quantify levels of DNA corresponding to the various regions of *INO1* (see panel **A**) bound to sumoylated proteins. Shown are the relative fold changes in the occupancy of sumoylated proteins associated with the various regions of *INO1* at the indicated times after induction relative to the uninduced samples. Results are the means ± SEM of five biological replicates. Asterisks indicate a significant difference relative to uninduced as determined by a Student’s paired *t*-test. *p < 0.05, **p < 0.01, ***p < 0.001.

Since the C-terminal domain of Ulp1 (residues 340–621) possesses both SUMO binding and desumoylase activity, we examined the effects of removing this domain of Ulp1 from the NPC on the sumoylation state of *INO1* bound proteins. To test this, we examined SUMO occupancy along the induced *INO1* locus in the *ulp1Δ_1__–__340_* mutant. In this mutant, we observed an increase in sumoylation of proteins in regions A and B of the *INO1* gene (adjacent to and containing the transcriptional start site) after induction (Figure 8B) similar to that seen in WT cells (Figure 1B). However unlike WT cells, the *ulp1Δ_1__–__340_* mutant showed no decrease in sumoylation in the GRS1 region and generally higher levels of protein sumoylation within *INO1* ORF (Figure 1B) where we detected Ulp1 binding in WT cells (Figure 4B). These data led us to conclude that Ulp1, within the context of the NPCs, functions to bind and desumoylate proteins associated with the induced *INO1* GRS1 and ORF.

## Discussion

Numerous observations have established that the spatial organization of the yeast genome is dynamic, and the positioning of numerous yeast genes within the nucleoplasm has been shown to be altered by their expression status. For example, transcriptional activation of the *INO1* gene, induced by a lack of inositol, is accompanied by its relocalization from the nuclear interior to an NPC. Here we report that these events are dependent on specific regulators of sumoylation, suggesting a role for sumoylation in the expression of the *INO1* gene and its targeting to the NPC. Conditions that induce expression of *INO1* lead to changes in the sumoylation of proteins associated with the *INO1* gene. These sumoylation events are largely mediated by the SUMO ligase Siz2, and, importantly, Siz2 is essential for the relocalization of activated *INO1* from the nucleoplasm to the NE. Concomitant with relocalization, *INO1* also interacts with NPC-associated Ulp1. Our data suggest that this interaction desumoylates *INO1*-associated proteins and is required for both targeting of the *INO1* locus to an NPC and normal induction of *INO1* expression. These results imply that a cycle of sumoylation and NPC-associated desumoylation contribute to *INO1* targeting to the NPC and its expression.

The observation that a gene locus relocalizes from the nucleoplasm to an NPC following activation was first described in yeast for the *INO1* gene, and it represents one of the most well studied of a growing list of genes exhibiting this behavior (Egecioglu and Brickner, 2011; Randise-Hinchliff and Brickner, 2016). Several factors required for *INO1* targeting to the NPC have been identified. Two cis-acting DNA elements 5′ to the *INO1* ORF are involved in *INO1* recruitment to NPCs following long-term repression (Ahmed et al., 2010; Light et al., 2010; Randise-Hinchliff et al., 2016). Both sites bind TFs, GRS-I binds Put3 and GRS-II binds Cbf1, and each TF is required for directing GRS-containing DNA elements to NPCs (Shetty and Lopes, 2010; Brickner and Brickner, 2012; Randise-Hinchliff et al., 2016; Brickner et al., 2019). Notably, while TFs may be required for NPC association of activated genes (Randise-Hinchliff et al., 2016; Brickner et al., 2019), RNA polymerase II-mediated transcription is not necessarily required, as shown for both *GAL1* and *INO1* (Schmid et al., 2006; Brickner et al., 2007, 2016).

The functions of Put3 and Cbf1 are not unique as various other TFs, including those functioning in inducible and constitutive expression, or acting as transcriptional regulators (including repressors), also have the potential to target genes to NPCs (Randise-Hinchliff et al., 2016; Brickner et al., 2019). Whether these various factors interact directly or indirectly with Nups is not clear, but it does appear that specific subsets of Nups are required for genes to interact with NPCs. For example, the binding of multiple TFs to NPCs requires Nup2 and Nup100 (Dilworth et al., 2005; Brickner et al., 2019). These and additional Nups positioned on the nucleoplasmic face of NPCs, including Nup60 and Nup1, have been linked to the NPC association of induced *INO1* (Brickner et al., 2007; Ahmed et al., 2010; Light et al., 2010). Of note, this function of Nup1 appears to require its phosphorylation by Cdc28 (Brickner et al., 2007; Brickner and Brickner, 2010). This and related observations support the idea that NPC association of activated *INO1* is cell cycle regulated, being lost during S-phase and reestablished during G2/M-phase where it primarily resides until the following S-phase (Brickner and Brickner, 2010).

**FIGURE 9:**
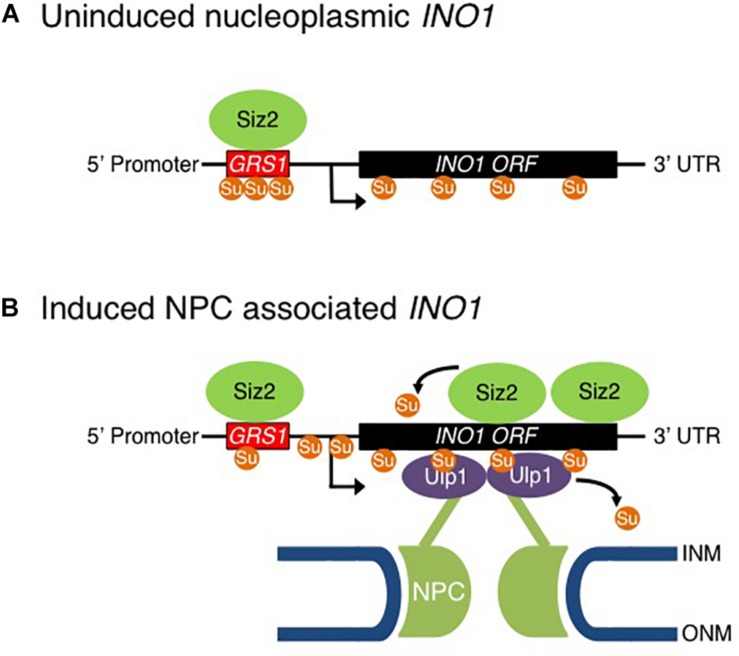
Model for Sumo-mediated recruitment of *INO1* to the NPC. Shown is model for the proposed role of sumoylation in the NPC targeting and transcriptional activation of the *INO1* gene. **(A)** In a repressed state, *INO1* is positioned in the nucleoplasm. **(B)** Reducing levels of inositol in the medium leads to recruitment of Siz2 to the *INO1* locus and increased Siz2-mediated sumoylation of *INO1*-associated proteins. These events are required for the targeting of the *INO1* locus to the NPC where it interacts with NPC-associated Ulp1. Ulp1 supports the association of *INO1* with the NPC and functions to desumoylation of *INO1* bound proteins in regions containing the GRS1 and the ORF. Desumoylation of *INO1*-associated proteins is proposed to promote *INO1* transcription.

Built upon the various requirements previously established for the expression and NPC-targeting of *INO1*, our data have led us to conclude that a cycle of sumoylation and desumoylation is essential for the expression and NPC targeting of activated *INO1*. We envisage a model for these processes that includes multiple steps that we assume initiate in the nucleoplasm (Figure 9). Prior to induction, Siz2 is bound to the *GRS1*-containing region of *INO1* (Supplementary Figure S2B) and Siz2-dependent sumoylation of proteins associated with the ORF is detected (Supplementary Figure S1C). Following induction, Siz2 binding increases within the *INO1* ORF (Figure 3B), as does sumoylation of targets associated with the 5′ region of the locus, including the *INO1* transcriptional start site (Figure 1, regions A and B). These increases in sumoylation are Siz2 dependent (Supplementary Figure S1C). While we have yet to identify the exact targets of Siz2 sumoylation prior to or following activation of INO1, considering their location within the *INO1* gene, it seems likely that these would include TFs that function in *INO1* targeting to the NPC, including Put3, which contains consensus sumoylation sites (Zhao et al., 2014), and Cbf1, which has previously been shown to be sumoylated (Wohlschlegel et al., 2004; Denison et al., 2005).

Siz2-mediated sumoylation events at the *INO1* locus may perform several functions. In some contexts, sumoylation of TFs and histones has been shown to contribute to transcriptional repression; whereas, in others, sumoylation has been linked to activation of gene transcription (Rosonina et al., 2010; Chymkowitch et al., 2015). Interestingly, previous reports have also implicated sumoylation in both repression and activation of certain genes, suggesting that the effects of sumoylation at a given gene are dependent on the target proteins bound to the locus. For example, analysis of the expression of the inducible *GAL1* gene suggests that it is maintained in a repressed state by sumoylation of two corepressors, Tup1 and Ssn6, that are desumoylated upon activation, reportedly by Ulp1 (Smith and Johnson, 2000; Zhang and Reese, 2004; Texari et al., 2013). Paradoxically, activation of the *GAL1* gene is also accompanied by the recruitment of the SUMO conjugating enzyme Ubc9 to the gene locus and increased levels of SUMO-modified proteins within the promoter region (Rosonina et al., 2010).

Similarly, activation of *INO1* is accompanied by both increased and decreased levels of bound SUMO-modified proteins at distinct regions of the gene (Figure 1B). In addition to increased sumoylation in regions A and B containing the transcriptional start site, *INO1* induction is accompanied by decreased sumoylation levels in the Put3-binding GRS1 region (Figure 1). These events raise the possibility that desumoylation of proteins associated with the GRS1 region contribute to increased *INO1* expression. In this regard, increased levels of Siz2-dependent sumoylation within the *INO1* gene observed in the *ulp1Δ_1__–__340_* mutant (Figure 8B) are coincident with reduced levels of *INO1* mRNA accumulation following induction (Figure 5B). Whether Siz2 sumoylation may play a repressive role in *INO1* expression will require further analysis.

Siz2 is required for targeting the activated *INO1* locus to the NE (Figure 2B). This could involve Siz2 directly mediating the binding of *INO1* to Nups. However, several observations have led us to conclude that Siz2-mediated sumoylation events direct *INO1* relocation to the NPC, including, for example, sumoylation of proteins bound to regions of *INO1* such as those near the transcriptional start site (Figure 1B). We speculate that the SUMO polypeptide may function as the NPC targeting signal. This idea is consistent with our observation that Ulp1, a SUMO binding protein, is required for the NE localization of *INO1* (Figure 5A). Specifically, we showed that the C-terminal domain of Ulp1, which contains SUMO binding sites within the catalytic pocket and a SIM domain, when alone anchored to the NPC, is sufficient for the accumulation of the activated *INO1* gene at the NPC (Figure 7D). Moreover, Ulp1 binds to the *INO1* ORF and is required for the desumoylation of Siz2-mediated sumoylation sites positioned within the ORF (Figures 4B, 8B). Finally, we observed that a *ulp1*^*CSDN*^ mutant protein, which exhibits reduced SUMO binding and no isopeptidase activity (Elmore et al., 2011) but binds to the NPC and competes with endogenous Ulp1 for NPC-binding (Figure 6B), also inhibits NE localization of *INO1* (Figure 6C). Each of these observations is consistent with a role for SUMO, and its association with Ulp1, in the targeting of *INO1* to the NPC (see Figure 9).

The requirement for NPC-bound Ulp1 in the targeting of activated *INO1* to the NPC provides further insight into previously described defects associated with certain *nup* mutations, including several encoding Nups positioned on the nucleoplasmic face of the NPC (Ahmed et al., 2010; Light et al., 2010). These include Nup60 and Nup2, both of which physically and functionally interact with Ulp1 (Zhao et al., 2004; Srikumar et al., 2013) and have been shown to play a role in the association of Ulp1 with the NPC. Here we have shown that the *INO1* localization defects associated with *nup60* and *nup2* null mutants can be rescued by positioning the Ulp1 C-terminal catalytic domain at the NPC as part of a fusion protein with Nup53 (Nup53-ulp1^340–621^; Figure 7) suggesting the role Nup2 and Nup60 play in this process is to position Ulp1 at the NPC.

The positioning of Ulp1 at the NPC is also essential for normal expression of *INO1*. In cells where NPC association of Ulp1 is inhibited, such as in the *ulp1Δ_1__–__340_* mutant, levels of *INO1* mRNA are reduced (Figures 5A, 7D). Importantly, placing the Ulp1 C-terminal catalytic domain at the NPC using Nup53-ulp1^340–621^ fusion rescued *INO1* expression defects in an otherwise WT background, as well as in the *nup60* and *nup2* null mutants (Figures 7D,F).

Cumulatively, our observations support a model in which induction of *INO1* is followed by increased binding of Siz2 to regions within the *INO1* ORF and its 3′ end. We propose that the sumoylation events that arise from the Siz2 binding facilitate binding of the *INO1* locus to NPC-bound Ulp1 and desumoylation of ORF-associated targets. The continuous presence of both Siz2 and Ulp1 bound to the *INO1* ORF (during the 3 h period of induction examined) may support a cycle of sumoylation and desumoylation of as yet unidentified target proteins that retains these proteins and the associated *INO1* gene at the NPC. Furthermore, Ulp1 binding and desumoylation of proteins associated with the *GRS1* region are also predicted to facilitate *INO1* binding to the NPC and potentially facilitate *INO1* transcription. Interestingly, Ulp1 bound near the 3′-end of the *INO1* gene might facilitate desumoylation of *GRS1* associated proteins as a consequence of *INO1* gene looping, which has been previously shown to occur following induction (Kaderi et al., 2009). Such a mechanism could also support Siz2 sumoylation of proteins within the 5′ region of *INO1*. We envisage that these steps in the NPC targeting and expression of *INO1* are built upon other key requirements previously reported for these processes, including specific DNA sequences, TFs, nuclear transport factors, and Nups (Chen et al., 2007; Light and Brickner, 2013; Randise-Hinchliff and Brickner, 2016).

The concepts described here for SUMO-mediated regulation of *INO1* localization and expression are likely to apply to other inducible genes. Of note, previous observations made in the analysis of the *GAL1* gene revealed sumoylation processes that occur during its activation that parallel events we have observed for *INO1*. For example, induction of the *GAL1* gene is accompanied by sumoylation of associated proteins (Rosonina et al., 2010), and NPC association of activated *GAL1* was inhibited when Ulp1 association with NPCs was reduced (Texari et al., 2013). It will be of interest to further test the broader impact of sumoylation and NPC-associated desumoylation.

## Data Availability Statement

The raw data supporting the conclusions of this article will be made available by the authors, without undue reservation, to any qualified researcher.

## Author Contributions

NP, NS, and CP performed the experiments. All authors contributed to the conception and design of the study, manuscript revision, and read and approved the submitted version. Figures were assembled by NS, NP, and CP. RW wrote the first draft of the manuscript.

## Conflict of Interest

The authors declare that the research was conducted in the absence of any commercial or financial relationships that could be construed as a potential conflict of interest.

## Supplementary Material

FIGURE S1 Sumoylation of proteins at the uninduced *INO1* locus. **(A)** Diagram of the *INO1* locus and regions detected by ChIP analysis (see Figure 1A). **(B,C)** The graphs show the relative fold enrichment of SUMO modified proteins at each position along the *INO1* gene relative to the level of SUMO modified proteins at an intergenic region within chromosome V as assessed by ChIP. **B** shows ChIP results for uninduced WT samples while **C** shows results comparing uninduced WT samples (WT 0 h) to samples derived from *siz2Δ* cells at the indicated times after *INO1* induction. Results are the means ± SEM of at least three biological replicates.

FIGURE S2 Siz2 associates with the *GRS1* region of the uninduced *INO1* locus. **(A)** Diagram of the *INO1* locus and regions detected by ChIP analysis (see Figure 1A). The graph shows the relative fold enrichment of Siz2-PrA **(B)** or Ulp1-PrA **(C)** at each position along the *INO1* gene relative to the level at an intergenic region within chromosome V as assessed by ChIP. Results are the means ± SEM of at least three biological replicates.

FIGURE S3 Characterization of chromosomally encoded *ulp1* truncation mutants. **(A)** Shown are cells producing Ulp1-GFP or the indicated truncation mutants (ulp1Δ_1__–__150_-GFP, ulp1Δ_150__–__340_-GFP, and ulp1Δ_1__–__340_-GFP) encoded by integrated and *ULP1* promoter-controlled mutant genes. Camera exposure times are equivalent for each strain. Scale bars = 2 μm. **(B)** Truncation mutants showed similar expression levels with that of full length Ulp1-GFP. Whole cell lysates of the indicated strains were tested by Western blotting with an anti-GFP antibody. For the loading control, the levels of Gsp1 were tested with an anti-Gsp1p antibody. **(C)** Whole cell lysates, derived from cultures of the indicated strains, were examined by Western blotting using an anti-Smt3 (SUMO) or an anti-Gsp1 (loading control) antibody. The positions of molecular mass markers are indicated in kilodaltons.

FIGURE S4 Growth of strains producing ulp1^*CSDN*^ and Nup53-ulp1^341–621^. **(A)** WT cells transformed with an empty plasmid (pEMPTY), a plasmid encoding WT Ulp1 (p*ULP1*), or a plasmid encoding the ulp1^*CSDN*^ mutant (p*ulp1*^*CSDN*^) were grown to mid-log phase in synthetic drop out liquid culture. Tenfold serial dilutions of each culture were made and cells from each dilution spotted onto synthetic drop out plates. Total cells plated ranged between 10^5^ and 10^2^ cells per spot. Plates were then incubated at 30°C for 2 days prior to imaging. **(B)** Cell lysates, derived from the same cultures described in **A**, were examined by Western blotting using an anti-Smt3 (SUMO) or an anti-Gsp1 (loading control) antibody. The positions of molecular mass markers are indicated in kilodaltons. **(C)** WT and *NUP53-ulp1^341–621^* cells were grown in YPD liquid culture to mid-log phase and cells from each culture analyzed as described in **A**.

TABLE S1 Yeast strains used in this study.
