# Supplementary figures and images for: Recruitment of an Activated Gene to the Yeast Nuclear Pore Complex Requires Sumoylation
Source: Front Genet. 2020 Mar 6;11:174. doi: 10.3389/fgene.2020.00174 (PMC7067905; doi:10.3389/fgene.2020.00174)

Figure S1

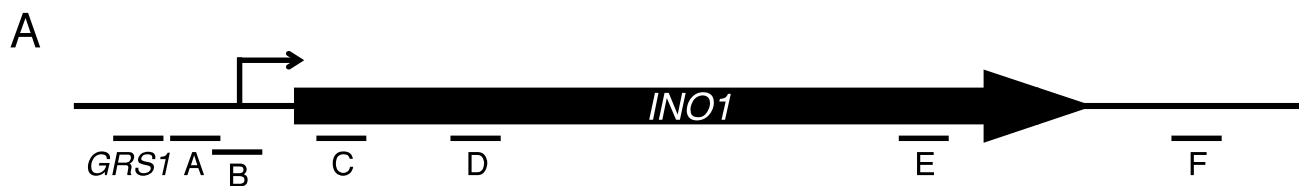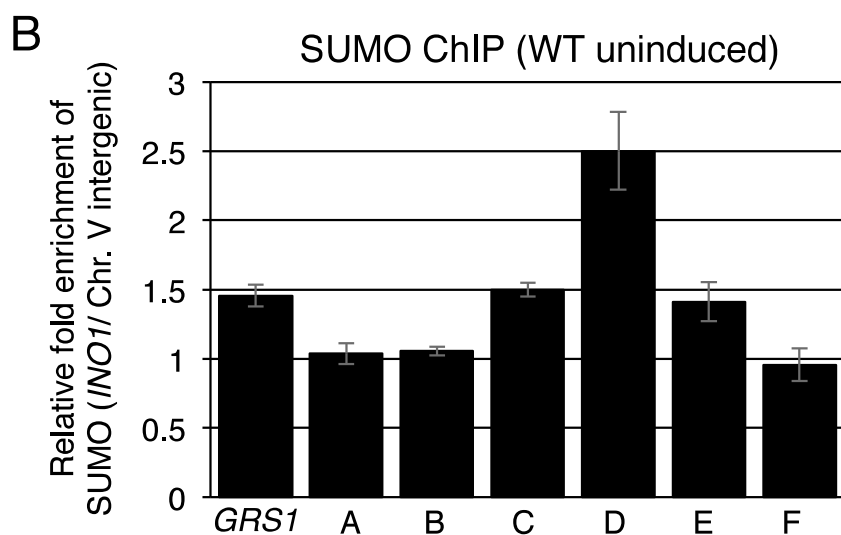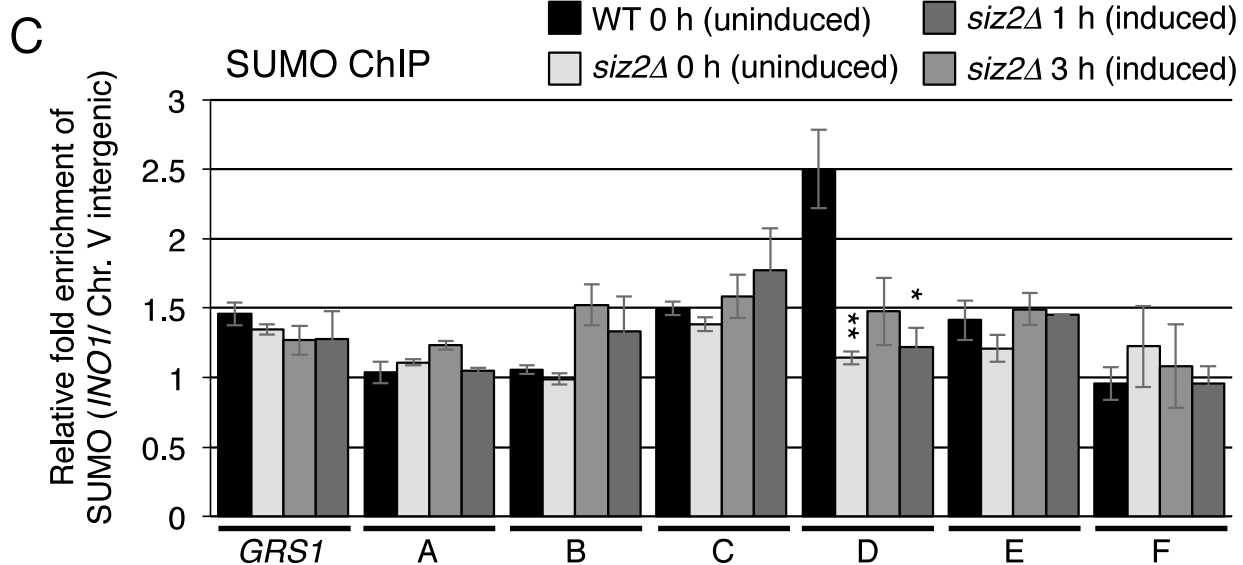

Supplement: FIGURE S1 — Sumoylation of proteins at the uninduced INO1 locus. (A) Diagram of the INO1 locus and regions detected by ChIP analysis (see Figure 1A). (B,C) The graphs show the relative fold enrichment of SUMO modified proteins at each position along the INO1 gene relative to the level of SUMO modified proteins at an intergenic region within chromosome V as assessed by ChIP. B shows ChIP results for uninduced WT samples while C shows results comparing uninduced WT samples (WT 0 h) to samples derived from siz2Δ cells at the indicated times after INO1 induction. Results are the means ± SEM of at least three biological replicates. [file Image_1.pdf]

Figure S2

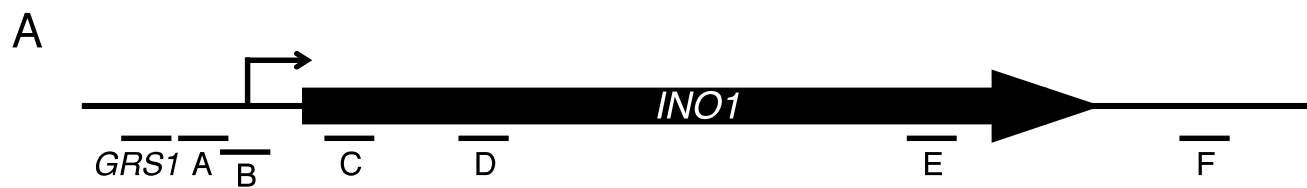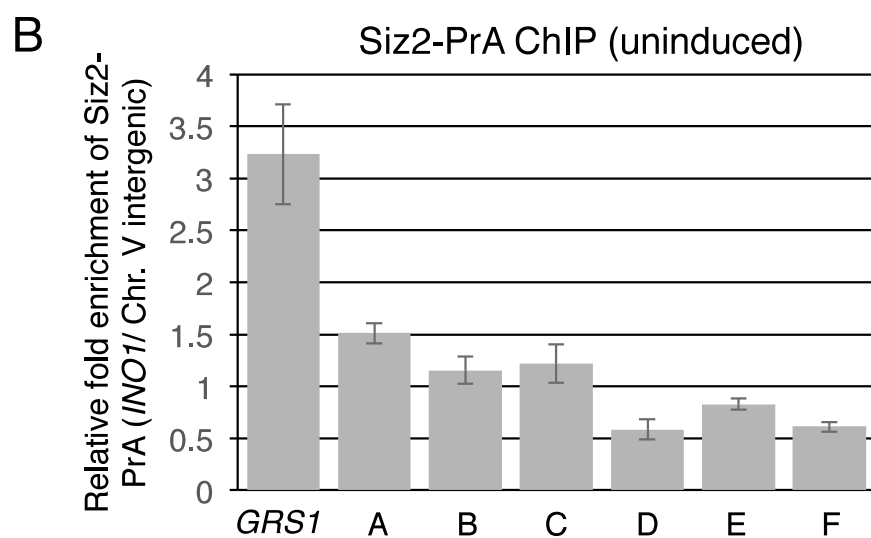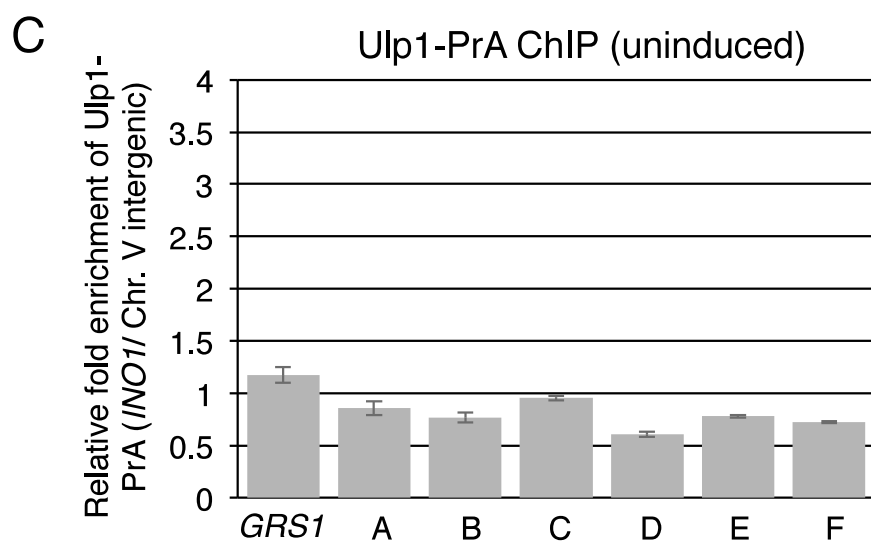

Supplement: FIGURE S2 — Siz2 associates with the GRS1 region of the uninduced INO1 locus. (A) Diagram of the INO1 locus and regions detected by ChIP analysis (see Figure 1A). The graph shows the relative fold enrichment of Siz2-PrA (B) or Ulp1-PrA (C) at each position along the INO1 gene relative to the level at an intergenic region within chromosome V as assessed by ChIP. Results are the means ± SEM of at least three biological replicates. [file Image_2.pdf]

Figure S3

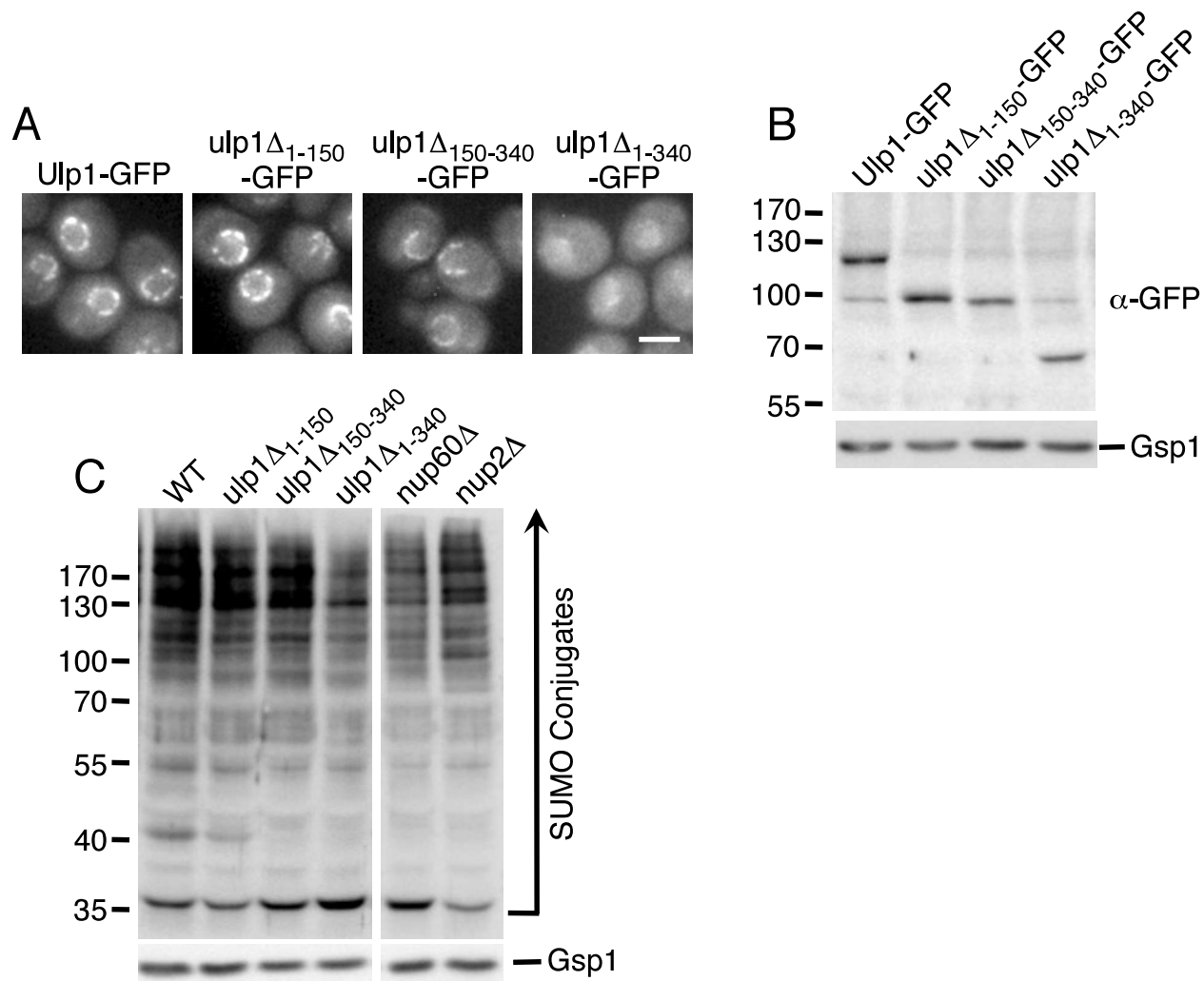

Supplement: FIGURE S3 — Characterization of chromosomally encoded ulp1 truncation mutants. (A) Shown are cells producing Ulp1-GFP or the indicated truncation mutants (ulp1Δ1–150-GFP, ulp1Δ150–340-GFP, and ulp1Δ1–340-GFP) encoded by integrated and ULP1 promoter-controlled mutant genes. Camera exposure times are equivalent for each strain. Scale bars = 2 μm. (B) Truncation mutants showed similar expression levels with that of full length Ulp1-GFP. Whole cell lysates of the indicated strains were tested by Western blotting with an anti-GFP antibody. For the loading control, the levels of Gsp1 were tested with an anti-Gsp1p antibody. (C) Whole cell lysates, derived from cultures of the indicated strains, were examined by Western blotting using an anti-Smt3 (SUMO) or an anti-Gsp1 (loading control) antibody. The positions of molecular mass markers are indicated in kilodaltons. [file Image_3.pdf]

Figure S4

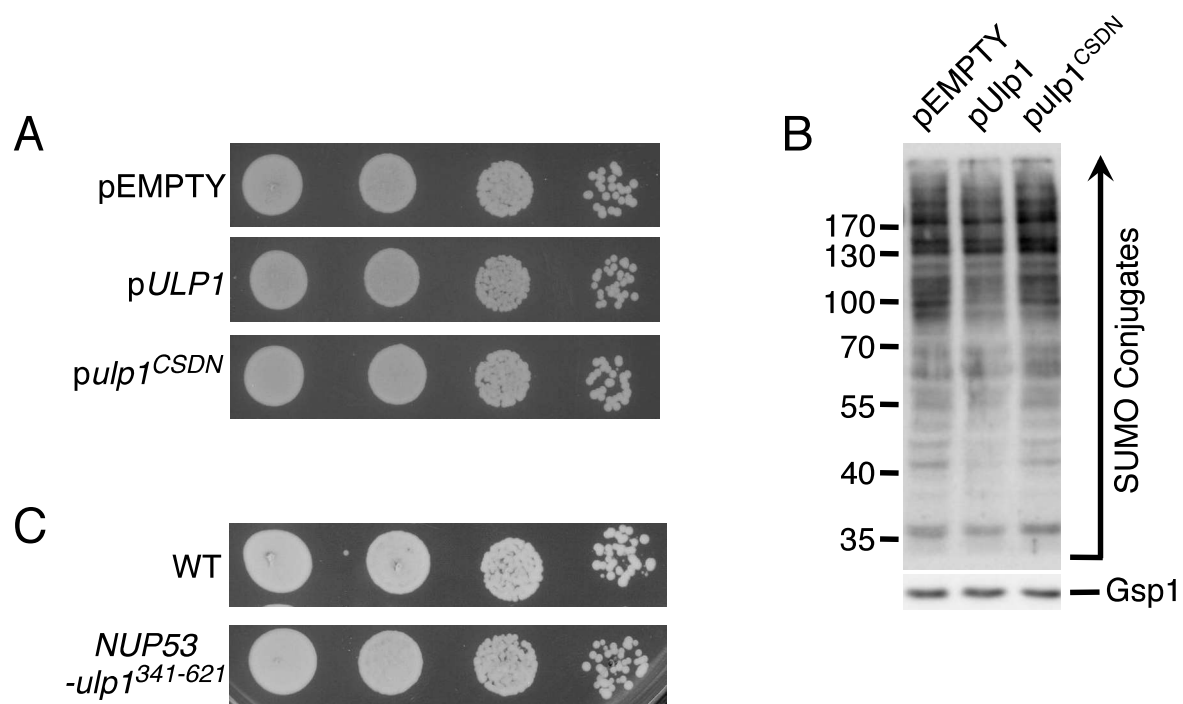

Supplement: FIGURE S4 — Growth of strains producing ulp1CSDN and Nup53-ulp1341–621. (A) WT cells transformed with an empty plasmid (pEMPTY), a plasmid encoding WT Ulp1 (pULP1), or a plasmid encoding the ulp1CSDN mutant (pulp1CSDN) were grown to mid-log phase in synthetic drop out liquid culture. Tenfold serial dilutions of each culture were made and cells from each dilution spotted onto synthetic drop out plates. Total cells plated ranged between 105 and 102 cells per spot. Plates were then incubated at 30°C for 2 days prior to imaging. (B) Cell lysates, derived from the same cultures described in A, were examined by Western blotting using an anti-Smt3 (SUMO) or an anti-Gsp1 (loading control) antibody. The positions of molecular mass markers are indicated in kilodaltons. (C) WT and NUP53-ulp1341–621 cells were grown in YPD liquid culture to mid-log phase and cells from each culture analyzed as described in A. [file Image_4.pdf]
