# Supplementary material for: Recruitment of an Activated Gene to the Yeast Nuclear Pore Complex Requires Sumoylation
Source: Front Genet. 2020 Mar 6;11:174. doi: 10.3389/fgene.2020.00174 (PMC7067905; doi:10.3389/fgene.2020.00174)
Supplement: TABLE S1 — Yeast strains used in this study. [file Table_1.DOCX]

| **Strain Name** | **Genotype** | **Source** |
| --- | --- | --- |
| *YEF473A | *MAT***a** *ura3-52 lys2-801 leu2-∆1 his3-∆200 trp1-∆63* | Bi and Pringle, 1996 |
| NY109 | *MAT***a** *ura3-52 lys2-801 leu2-∆1 his3-∆200::lacI-GFP-HIS3 INO1::lacO(256)-TRP1 NUP49-mRFP-hph* | This Study |
| NPY1202 | *MAT***a** *ura3-52 lys2-801 leu2-∆1 his3-∆200::lacI-GFP-HIS3 INO1::lacO(256)-TRP1 NUP49-mRFP-HPH siz1Δ::KAN* | This Study |
| NPY1203 | *MAT***a** *ura3-52 lys2-801 leu2-∆1 his3-∆200::lacI-GFP-HIS3 INO1::lacO(256)-TRP1 NUP49-mRFP-HPH siz2Δ::KAN* | This Study |
| NPY1010 | *MAT***a** *ura3-52 lys2-801 leu2-∆1 his3-∆200 trp1-∆63 SIZ2-PrA-HIS3* | This Study |
| CPY4245 | *MAT***a** *ura3-52 lys2-801 leu2-∆1 his3-∆200 trp1-∆63 siz2Δ::KAN* | This Study |
| NPY1101 | *MAT***a** *ura3-52 lys2-801 leu2-∆1 his3-∆200 trp1-∆63 ULP1-PrA-HIS3* | This Study |
| NY259 | *MAT***a** *ura3-52 lys2-801 leu2-∆1 his3-∆200 trp1-∆63 ulp1Δ_1-150_-NAT* | This Study |
| CPY4198 | *MAT***a** *ura3-52 lys2-801 leu2-∆1 his3-∆200 trp1-∆63 ulp1Δ_150-340_-NAT* | This Study |
| CPY4182 | *MAT***a** *ura3-52 lys2-801 leu2-∆1 his3-∆200 trp1-∆63 ulp1Δ_1-340_-KAN* | This Study |
| CPY4201 | *MAT***a** *ura3-52 lys2-801 leu2-∆1 his3-∆200 trp1-∆63 ULP1-GFP-HIS* | This Study |
| CPY4202 | *MAT***a** *ura3-52 lys2-801 leu2-∆1 his3-∆200 trp1-∆63 ulp1Δ_150-340_-GFP-HIS* | This Study |
| CPY4203 | *MAT***a** *ura3-52 lys2-801 leu2-∆1 his3-∆200 trp1-∆63 ulp1Δ_1-340_-GFP-HIS* | This Study |
| CPY4204 | *MAT***a** *ura3-52 lys2-801 leu2-∆1 his3-∆200 trp1-∆63 ulp1Δ_1-340_-GFP-HIS* | This Study |
| NY336 | *MAT***a** *ura3-52 lys2-801 leu2-∆1 his3-∆200::lacI-GFP-HIS3 INO1::lacO(256)-TRP1 NUP49-mRFP-HPH ulp1Δ_1-150_-NAT* | This Study |
| NY337 | *MAT***a** *ura3-52 lys2-801 leu2-∆1 his3-∆200::lacI-GFP-HIS3 INO1::lacO(256)-TRP1 NUP49-mRFP-HPH ulp1Δ_150-340_-NAT* | This Study |
| CPY4191 | *MAT***a** *ura3-52 lys2-801 leu2-∆1 his3-∆200::lacI-GFP-HIS3 INO1::lacO(256)-TRP1 NUP49-mRFP-HPH ulp1Δ_1-340_-KAN* | This Study |
| CPY200 | *MAT***a** *ura3-52 lys2-801 leu2-∆1 his3-∆200::lacI-GFP-HIS3 INO1::lacO(256)-TRP1 NUP49-mRFP-HPH ulp1Δ_1-340_-KAN pRS315* | This Study |
| CPY201 | *MAT***a** *ura3-52 lys2-801 leu2-∆1 his3-∆200::lacI-GFP-HIS3 INO1::lacO(256)-TRP1 NUP49-mRFP-HPH ulp1Δ_1-340_-KAN pRS315.ULP1* | This Study |
| CPY202 | *MAT***a** *ura3-52 lys2-801 leu2-∆1 his3-∆200::lacI-GFP-HIS3 INO1::lacO(256)-TRP1 NUP49-mRFP-hph pRS315* | This Study |
| CPY203 | *MAT***a** *ura3-52 lys2-801 leu2-∆1 his3-∆200::lacI-GFP-HIS3 INO1::lacO(256)-TRP1 NUP49-mRFP-hph pRS315-ULP1* | This Study |
| CPY204 | *MAT***a** *ura3-52 lys2-801 leu2-∆1 his3-∆200 trp1-∆63 pRS315-ULP1-GFP* | This Study |
| CPY205 | *MAT***a** *ura3-52 lys2-801 leu2-∆1 his3-∆200 trp1-∆63 pRS315-ulp1^CSDN^-GFP* | This Study |
| CPY206 | *MAT***a** *ura3-52 lys2-801 leu2-∆1 his3-∆200 trp1-∆63 ULP1-mCherry-NAT pRS315-ULP1-GFP* | This Study |
| CPY207 | *MAT***a** *ura3-52 lys2-801 leu2-∆1 his3-∆200 trp1-∆63 ULP1-mCherry-NAT pRS315-ulp1^CSDN^-GFP* | This Study |
| CPY209 | *MAT***a** *ura3-52 lys2-801 leu2-∆1 his3-∆200::lacI-GFP-HIS3 INO1::lacO(256)-TRP1 NUP49-mRFP-HPH pRS315* | This Study |
| CPY209 | *MAT***a** *ura3-52 lys2-801 leu2-∆1 his3-∆200::lacI-GFP-HIS3 INO1::lacO(256)-TRP1 NUP49-mRFP-HPH pRS315.ULP1-GFP* | This Study |
| CPY210 | *MAT***a** *ura3-52 lys2-801 leu2-∆1 his3-∆200::lacI-GFP-HIS3 INO1::lacO(256)-TRP1 NUP49-mRFP-HPH pRS315.ulp1^CSDN^-GFP* | This Study |
| CPY211 | *MAT***a** *ura3-52 lys2-801 leu2-∆1 his3-∆200::lacI-GFP-HIS3 INO1::lacO(256)-TRP1 NUP49-mRFP-HPH pRS315.ULP1* | This Study |
| CPY212 | *MAT***a** *ura3-52 lys2-801 leu2-∆1 his3-∆200::lacI-GFP-HIS3 INO1::lacO(256)-TRP1 NUP49-mRFP-HPH pRS315.ulp1^CSDN^* | This Study |
| NY347 | *MAT*a *ura3-52 lys2-801 leu2-∆1 his3-∆200::lacI-GFP-HIS3 INO1::lacO(256)-TRP1 NUP49-mRFP-HPH nup53Δ::KAN nup60Δ-URA3* | This Study |
| NPY2076 | *MAT*a *ura3-52 lys2-801 leu2-∆1 his3-∆200::lacI-GFP-HIS3 INO1::lacO(256)-TRP1 NUP49-mRFP-HPH nup53Δ::KAN nup2Δ-URA3* | This Study |
| NPY2001 | *MAT*a *ura3-52 lys2-801 leu2-∆1 his3-∆200::lacI-GFP-HIS3 INO1::lacO(256)-TRP1 NUP49-mRFP-hph ULP1::P_ULP1_-NUP53-ulp1^340-621^-NAT nup53Δ::KAN* | This Study |
| NPY2013 | *MAT*a *ura3-52 lys2-801 leu2-∆1 his3-∆200::lacI-GFP-HIS3 INO1::lacO(256)-TRP1 NUP49-mRFP-HPH nup53Δ::KAN nup60Δ-URA3 ULP1::P_ULP1_-NUP53-ulp1^340-621^-NAT* | This Study |
| NPY2079 | *MAT*a *ura3-52 lys2-801 leu2-∆1 his3-∆200::lacI-GFP-HIS3 INO1::lacO(256)-TRP1 NUP49-mRFP-HPH nup53Δ::KAN nup2Δ-URA3 ULP1::P_ULP1_-NUP53-ulp1^340-621^-NAT* | This Study |
| CPY4183 | *MAT***a** *ura3-52 lys2-801 leu2-∆1 his3-∆200 trp1-∆63 nup60Δ::HPH* | This Study |
| CPY4184 | *MAT***a** *ura3-52 lys2-801 leu2-∆1 his3-∆200 trp1-∆63 nup2Δ::HPH* | This Study |
| NPY2032 | *MAT***a** *ura3-52 lys2-801 leu2-∆1 his3-∆200 trp1-∆63 nup53Δ::KAN ULP1::P_ULP1_-NUP53-ulp1^340-621^-GFP-HIS* | This Study |
| CPY4185 | *MAT***a** *ura3-52 lys2-801 leu2-∆1 his3-∆200 trp1-∆63 nup60Δ::HPH nup53Δ::KAN ULP1::P_ULP1_-NUP53-ulp1^340-621^-GFP-HIS* | This Study |
| CPY4186 | *MAT***a** *ura3-52 lys2-801 leu2-∆1 his3-∆200 trp1-∆63 nup2Δ::HPH nup53Δ::KAN ULP1::P_ULP1_-NUP53-ulp1^340-621^-GFP-HIS* | This Study |

*All strains are derived from YEF473A
